# Un faux kyste pancréatique mimant une tumeur kystique et doublement compliqué de compression digestive et d'hémorragie: la pancréatite aiguë, la saga continue

**DOI:** 10.11604/pamj.2014.18.221.4730

**Published:** 2014-07-16

**Authors:** Taoufiq Ameuraoui, Baderedine Alami, Meryem Boubbou, Mustapha Maaroufi, Imane Kamaoui, Siham Tizniti

**Affiliations:** 1 Département de Radiologie, CHU Hassan II, Fès, Maroc

**Keywords:** Faux kyste, pancréas, tumeur, hémorragie, pancréatite aigue, pseudocyst, pancreas, tumor, bleeding, acute pancreatitis

## Abstract

L'histoire naturelle des pancréatites aigues nécrotico-hémorragiques est parfois caractérisée par la survenue imprévisible de plusieurs complications. La formation de pseudos kystes constitue un tournant évolutif important lors de sa survenue. Son évolution est imprévisible pouvant aller de la simple résorption spontanée à la survenue de complications gravissimes. Les principales complications observées sont l'obstruction, la surinfection et l'hémorragie. Nous rapportons le cas d'une pancréatite aigue lithiasique compliquée d'un faux kyste de pancréas compressif et d'un faux anévrysme artériel. L'intérêt de cet observation réside d'une part dans l'originalité de la présentation clinique, du fait que l'histoire naturelle de cette affection n'est pas toujours connue, ce qui pose parfois des difficultés diagnostic et justifie une discussion pluridisciplinaire, et d'autre part dans la rareté des cas publiés avec, à la fois, des complications hémorragique,et compressive: digestive et canalaires biliaires.

## Introduction

L'histoire naturelle des pancréatites aigues nécrotico-hémorragiques est parfois caractérisée par la survenue imprévisible de plusieurs complications. La formation de pseudo kystes constitue un tournant évolutif important lors de sa survenue. Sa fréquence est estimée entre 5 et 15% des cas dans la littérature [1, 2]. Son évolution est imprévisible pouvant aller de la simple résorption spontanée à la survenue de complications gravissimes. Les principales complications observées sont l'obstruction, la surinfection et l'hémorragie. Les complications hémorragiques sont le plus souvent en rapport avec la constitution d'un faux anévrysme [3]. Leur prise en charge est complexe et doit être multidisciplinaire. Nous rapportons le cas d'une pancréatite aigue lithiasique compliquée d'un faux kyste de pancréas compressif et d'un faux anévrysme artériel.

## Patient et observation

**Figure 1:**
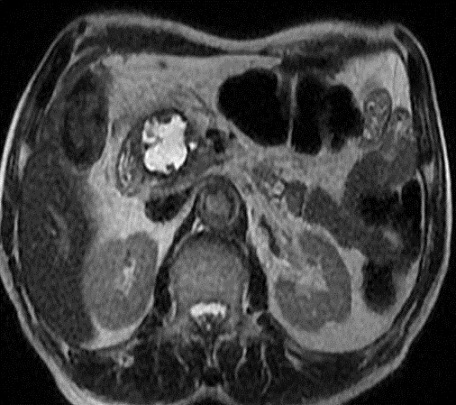
IRM en pondération T2 confirme le caractère kystique de la masse uniloculaire, et la dilatation du wirsung qui ne présente pas de communication avec la lésion

**Figure 2:**
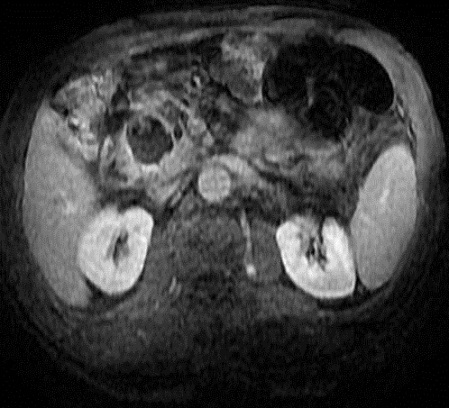
IRM en pondération T1 après injection de produit paramagnétique montre le contenu hétérogène et la paroi épaissie de la masse kystique

**Figure 3:**
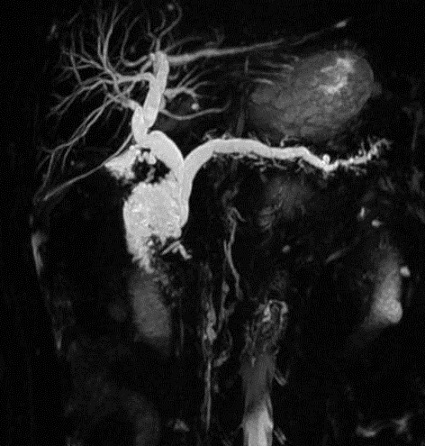
Cholongio IRM montre la dilatation bicanalaire en amont de la masse kystique

Mr B. A, âge 71 ans, aux antécédents de cholécystectomie il ‘y a 7 mois, il s'est présenté pour ictère choléstatique. A l'examen clinique, il présentait un ictère cutanéo-muqueux cholestatique, associé à une altération de l’état générale (5kg/ 3 mois). La biologie a montré un bilan de cholestase et de cytolyse élevé. Une échographie, faite à titre externe, parle d'une masse kystique de la tête du pancréas avec dilatation bicanalaire, suite à laquelle il a été adréssé chez nous au service pour complément IRM. L'IRM faite le 24/01/2011 montre une masse liquidienne uniloculaire de la tête du pancréas qui mesure 3 cm, à paroi épaissie qui prend le contraste, le contenu est hétérogène, avec dilatation bicanalaire (wirsung et VBP),sans individualisation de communication avec les canaux pancréatiques, pouvant être en rapport avec un cystadénome mucineux, cependant un adénocarcinome kystique est à évoquer aussi (Figure 1, Figure 2, Figure 3).

**Figure 4:**
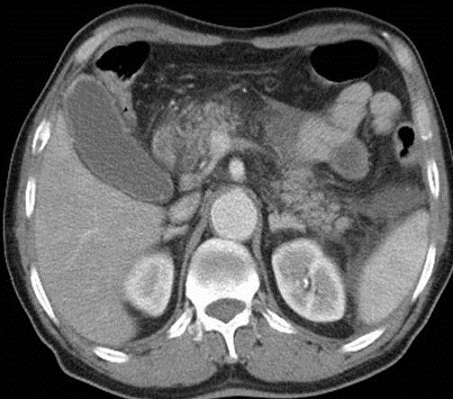
Scanner abdominal après injection du PDC iodé (B): coulées de nécrose au niveau de l'ACE et en para-rénal gauche en faveur d'une pancréatite stade E

La décision des chirurgiens était de programmer le patient pour duodéno-pancréatectomie céphalique,après avoir réaliser un scanner thoraco-abdomino-pelvien dans le cadre du bilan d'extension. Le scanner a conclu aux mêmes donnés de l'IRM et en l'absence d'extension locorégionale ou à distance. Sauf qu'on est surpris par la découverte sur le PACS d'un scanner fait le mois 05/2010 montrant des signes de pancréatite aigue stade E,avec présence de calculs vésiculaire et cholédocien sans individualisation de lésion kystique (Figure 4). Devant ce contexte,on a retenu le faux kyste de pancréas comme diagnostic final de cette masse kystique.

**Figure 5:**
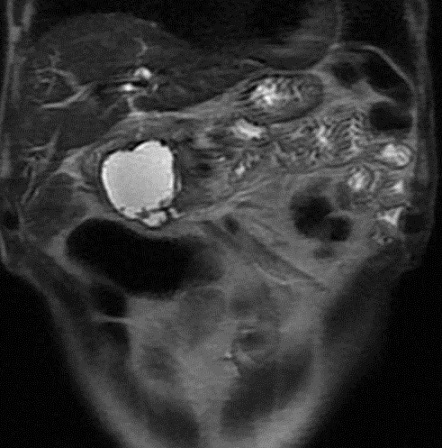
IRM de contrôle en pondération T2 montrant l'augmentation en taille du faux kyste venant au contact du duodénum qui est comprimé

**Figure 6:**
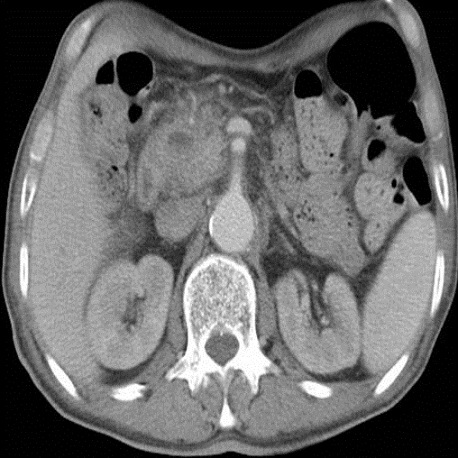
TDM abdominal après injection du PDC iodé réalisée après affaissement spontané du kyste montrant la régression en taille de ce dernier

Ce faux kyste est devenu compressif, avec installation d'ictère, de douleurs épigastriques, de vomissements alimentaires et dyspepsie. Une IRM de contrôle a été réalisée le mois 10/2011 objectivant la majoration en taille de la lésion kystique du pancréas (50mm versus 30 mm) sans signes d'atypie, avec persistance de la dilatation bicanalaire. Cette fois-ci les gastro-entérologues ont décidé de drainer cette collection par fibroscopie, mais le kyste s'est affaissé spontanément avant la réalisation du geste endoscopique (Figure 5, Figure 6).

**Figure 7:**
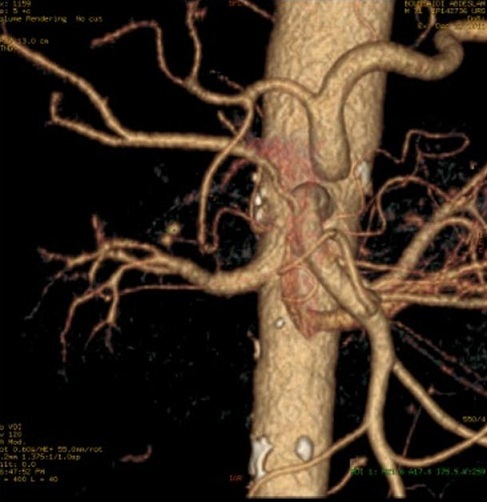
Angioscanner abdominal en reconstruction VR montrant la présence d'un faux anévrysme sur une branche de l'arcade duodéno-pancréatique

L’évolution ultérieure était marquée par l'apparition, le mois 12/2011, d'hématémèses et mélénas de grande abondance. Un angioscanner, réalisé le 22/12/2011, montre un blush artériel en rapport avec une rupture d'un faux anévrysme sur une arcade duodéno-pancréatique (Figure 7). le patient a été transféré vers un autre centre pour embolisation, un angioscanner refait a montré l'absence de blush et la thrombose du faux anévrysme.

## Discussion

Nous rapportons le cas d'un patient atteint de pancréatite d'origine biliaire dont l’évolution a été marquée par la survenue d'un syndrome compressif bilio-digestif et par la rupture d'un pseudoanévrisme de l'artère duodéno-pancréatique. L'intérêt de cet observation réside d'une part dans l'originalité de la présentation clinique, du fait que l'histoire naturelle de cette affection n'est pas toujours connue, ce qui pose parfois des difficultés diagnostic et justifie une discussion pluridisciplinaire, comme cela a été le cas dans notre observation, et d'autre part dans la rareté des cas publiés avec, à la fois, des complications hémorragique,et compressive: digestive et canalaires biliaires.

Le diagnostic d′un pseudo-kyste, apparaissant dans les suites d′une poussée de pancréatite aigue ne pose généralement aucun problème, sous réserve que le bilan initial (à 48 heures) de la pancréatite aigue réalisée au scanner ne montrait pas de formation kystique. Si ce premier bilan n′a pas été réalisé et que le patient est vu à distance de l′épisode aigu, le diagnostic peut être plus difficile. Ce d'autant que les lésions tumorales kystiques du pancréas peuvent être à l′origine de poussées de pancréatite [4, 5]. Notre patient a présenté un problème de diagnostic différentiel avec les autres étiologies des masses kystiques pancréatiques, d'o“ la nécessité d'une confrontation de toutes les sources d'information disponibles: histoire de la maladie, imagerie radiologique, voire parfois écho-endoscopie, et si nécessaire, analyse cytologique du liquide après ponction guidée à l'aiguille fine.

Le faux kyste correspond à l’évolution d'une collection liquidienne non infectée. Il complique environ 6% des pancréatites nécrotiques. Ces faux kystes se résorbent spontanément dans 50% des cas, même tardivement. Ils peuvent entraîner des compressions de voisinage, des hémorragies ou s'infecter secondairement [6]. Les complications intéressent donc environ 50% des pseudo-kystes de plus de 6 cm évoluant depuis plus de 6 semaines et ce sans facteur prédictif [7]. Les complications sont la douleur, la sensation de gêne, les compressions digestives, biliaires, du conduit pancréatique, les thromboses vasculaires le plus souvent veineuses, l′infection, l′hémorragie, le pseudo-anévrisme et la rupture du kyste.

Dans notre observation, on a rapporté deux complications: compression digestive des voies biliaires et duodénale, et hémorragique par rupture de faux anévrysme, dont la prise en charge nécessite une concertation multidisciplinaire faisant intervenir radiologues, gastro-entérologues, chirurgiens et réanimateurs. Le diagnostic de compression est le plus souvent fait au scanner. S′il s′agit d′une compression digestive, l′absorption de plusieurs verres d′eau juste avant l′acquisition permet de mieux délimiter la filière gastroduodénale et d′apprécier au mieux la compression. Si le bilan concerne les compressions canalaires biliaires ou pancréatiques. Ils seront au mieux analysées par la cholangio-pancréato-IRM. Le traitement est le plus souvent endoscopique ou chirurgical, la radiologie interventionnelle peut éventuellement avoir un rôle temporaire.

Le pseudoanévrisme est une complication rare des pseudokystes du pancréas, mais s'associe à une mortalité très élevée. Il peut survenir après une poussée de pancréatite aiguë ou au cours d'une pancréatite chronique. L'artère splénique est l'artère la plus souvent concernée, mais les artères gastroduodénale, pancréaticoduodénale, gastrique et hépatique peuvent également être concernées [8]. Les pseudoanévrismes se manifestent le plus souvent par une hémorragie: hématémèse et méléna en cas de rupture dans le tube digestif, hémorragie intrakystique en cas de rupture vers le pseudokyste ou hémopéritoine en cas de rupture intrapéritonéale. Ces hémorragies ont une mortalité élevée comprise entre 15% et 43% [9]. La survenue d'une hémorragie digestive chez un patient atteint de pancréatite chronique nécessite la réalisation d'une imagerie en urgence, permettant le diagnostic précoce de complications pseudoanévrismales. Le scanner multibarettes avec injection est l'examen initial de choix en raison de sa faisabilité et de sa bonne sensibilité diagnostique [10]. L'angiographie a également une excellente sensibilité diagnostique allant de 96 à 100% mais en raison de son caractère invasif, elle reste réservée aux indications thérapeutiques [11]. L’échoendoscopie digestive est probablement un examen fiable permettant le diagnostic de pseudoanévrysmes et leurs complications [12]. Aucune étude n'a comparé les performances diagnostiques de l’échoendoscopie dans ce contexte, par rapport aux autres techniques d'imagerie.

Le traitement actuel des pseudoanévrismes comporte souvent dans un premier temps une embolisation artérielle qui contrôle l'hémorragie dans 67 à 100% des cas [13]. L'embolisation est parfois une procédure d'attente, permettant de différer le geste chirurgical qui reste souvent nécessaire compte tenu du risque de récidive hémorragique [12].

## Conclusion

La survenue d'un pseudo-kyste au décours d'une pancréatite aigue est un évènement fréquent. Le rôle de l'imagerie est d'en faire le diagnostic positif, et de savoir évoquer les diagnostics différentiels, notamment les tumeurs kystiques pancréatiques qui nécessiteront une prise en charge différente. Il n'existe pas de facteur morphologique prédictif de l’évolution d'un pseudo-kyste; aussi la prise en charge thérapeutique doit tenir compte de la symptomatologie clinique. La prise en charge des pseudos kystes est multidisciplinaire (gastro-entérologique, chirurgicale et radiologique). Le rôle de la radiologie interventionnelle est important dans les complications vasculaires hémorragiques, digestives et infectieuses.

## References

[1] Baillie G (2004). Pancreatic pseudocysts (PartI). Gastrointest Endosc..

[2] Barthet M, Bugallo M, Moreira LS (1992). Traitement des pseudokystes après pancréatite aiguë: Étude rétrospective de 45 patients. Gastroenterol Clin Biol..

[3] Ben Moussa M, Feki MN, Baraket O, Chaari M, Saidani A, Bouchoucha S (2011). Un pseudo kyste pancréatique doublement compliqué d'infection et d'hémorragie. La Tunisie Médicale..

[4] Kim YH, Saini S, Sahani D, Hahn PF, Mueller PR, Auh YH (2005). Imaging diagnosis of cystic pancreatic lesions:pseudocyst versus nonpseudocyst. Radiographics..

[5] Sahani DV, Kadavigere R, Saokar A, Fernandez-del Castillo C, Brugge WR, Hahn PF (2005). Cystic pancreatic lesions: a simple imaging-based classification system for guiding management. Radiographics..

[6] Maury E, Lecesne R (2001). Comment et à quel moment établir la gravité d'une pancréatite aiguë?. Gastroenterol Clin Biol..

[7] Bruel JM, Aufort S, Zins M, Vivens F, Gallix B, Taourel P (2006). La prise en charge des pancréatites aiguës (PA): rôle de l'imagerie. Journées françaises de radiologie.

[8] Brouquet A, Lefevre JH, Terris B, Silvera S, Randone B, Soubrane O, Scatton O (2007). Un pseudo-kyste pancréatique responsable de trois complications hémorragiques simultanées. J Chir (Paris)..

[9] Bruce Stabile E, Samuel Wilson E, Haile Debas T (1983). Reduced Mortality From Bleeding Pseudocysts and Pseudoaneurysms Caused by Pancreatitis. Arch Surg..

[10] Balthazar EJ, Fisher LA (2001). Hemorrhagic complications of pancreatitis: radiologic evaluation with emphasis on CT imaging. Pancreatology..

[11] Balachandra S, Siriwardena AK (2005). Systemic appraisal of the management of the major vascular complications of pancreatitis. Am J Surg..

[12] Cattan P, Cuillerier E, Cellier C, Cuenod CA, Roche A, Landi B, Barbier JP (1999). Hemobilia caused by a pseudoaneurysm of the hepatic artery diagnosed by EUS. Gastrointest Endosc..

[13] De Perrot M, Berney T, Bühler L, Delgadillo X, Mentha G, Morel P (1999). Management of bleeding pseudoaneurysms in patients with pancreatitis. Br J Surg..

